# Parvoviruses of Aquatic Animals

**DOI:** 10.3390/pathogens13080625

**Published:** 2024-07-26

**Authors:** Frederick Kibenge, Molly Kibenge, Marco Montes de Oca, Marcos Godoy

**Affiliations:** 1 Department of Pathology and Microbiology, Atlantic Veterinary College, University of Prince Edward Island, Charlottetown, PE C1A 4P3, Canada; mkibenge@upei.ca; 2 Centro de Investigaciones Biológicas Aplicadas (CIBA), Puerto Montt 5480000, Chile; marco.montesdeoca@ciba.cl (M.M.d.O.); or marcos.godoy@uss.cl (M.G.); 3 Laboratorio de Biotecnología Aplicada, Facultad de Ciencias de la Naturaleza, Escuela de Medicina Veterinaria, Sede de la Patagonia, Universidad San Sebastián, Puerto Montt 5480000, Chile

**Keywords:** *Parvoviridae*, finfish, crustaceans, mollusks, pathogens, viruses, *Parvovirinae*, *Densovirinae*, *Hamaparvovirinae*, *Metalloincertoparvovirus*

## Abstract

Family *Parvoviridae* consists of small, non-enveloped viruses with linear, single-stranded DNA genomes of approximately 4-6 kilobases, subdivided into three subfamilies, *Parvovirinae*, *Densovirinae,* and *Hamaparvovirinae*, and unassigned genus *Metalloincertoparvovirus*. Parvoviruses of aquatic animals infect crustaceans, mollusks, and finfish. This review describes these parvoviruses, which are highly host-specific and associated with mass morbidity and mortality in both farmed and wild aquatic animals. They include Cherax quadricarinatus densovirus (CqDV) in freshwater crayfish in Queensland, Australia; sea star-associated densovirus (SSaDV) in sunflower sea star on the Northeastern Pacific Coast; Clinch densovirus 1 in freshwater mussels in the Clinch River, Virginia, and Tennessee, USA, in subfamily *Densovirinae*; hepatopancreatic parvovirus (HPV) and infectious hypodermal and hematopoietic necrosis virus (IHHNV) in farmed shrimp worldwide; *Syngnathid ichthamaparvovirus 1* in gulf pipefish in the Gulf of Mexico and parts of South America; tilapia parvovirus (TiPV) in farmed tilapia in China, Thailand, and India, in the subfamily *Hamaparvovirinae*; and *Penaeus monodon* metallodensovirus (PmMDV) in Vietnamese *P. monodon*, in unassigned genus *Metalloincertoparvovirus*. Also included in the family *Parvoviridae* are novel parvoviruses detected in both diseased and healthy animals using metagenomic sequencing, such as zander parvovirus from zander in Hungary and salmon parvovirus from sockeye salmon smolts in British Columbia, Canada.

## 1. Introduction

Members of the family *Parvoviridae* are small, non-enveloped viruses with linear, single-stranded (ss) DNA genomes of approximately 4–6 kb. The family is subdivided into three phylogenetically defined subfamilies and an unassigned genus (*Metalloincertoparvovirus*): *Parvovirinae*, *Densovirinae*, and *Hamaparvovirinae*. Viruses in *Parvovirinae* (11 genera) and *Densovirinae* (11 genera) are distinguished primarily by their ability to infect vertebrate hosts versus invertebrate hosts (insects, shrimp, and echinoderms), respectively. In contrast, those in the subfamily *Hamaparvovirinae* infect both vertebrate (two genera) and invertebrate (three genera) hosts, hence the subfamily name from the ancient Greek word meaning “together” [1]. Because of their small genome, parvoviruses require actively dividing host cells to transcribe their viral genome and are host- and tissue-specific. Some cause diseases that range from subclinical to lethal. A few require coinfection with helper viruses from other families [2]. To our knowledge, this is the first comprehensive review article describing all the reported aquatic animal parvoviruses and putting them in the context of terrestrial animal parvoviruses, both veterinary [3] and human parvoviruses [4], to add to the current knowledge on the family *Parvoviridae*.

Novel zander parvovirus from zander or pikeperch (*Sander lucioperca*), a freshwater fish in Hungary, potentially represents a new genus and a new species in the subfamily *Parvovirinae* [5]. The novel zander parvovirus was detected by viral metagenomics and PCR methods in fecal samples collected from fish showing no clinical signs, and the possibility of a dietary origin of the virus could not be excluded [5]. Zander parvovirus is possibly the third member in the subfamily *Parvovirinae* to infect an aquatic animal host. The novel salmon parvovirus from sockeye salmon smolts in British Columbia, Canada, [6] was the first to be reported but its full genome sequence is not yet publicly available, and its taxonomic location in the *Parvoviridae* family remains unassigned.. The second is *Crangon crangon* parvo-like virus 1 (CcPaLV 1) detected in European mollusks [7], and currently unassigned to a genus in the subfamily *Parvovirinae*.

Members of the subfamily *Densovirinae* can be highly host-specific and lethal [8]. Mass mortality in invertebrates is a well-characterized consequence of densovirus infection, with *Densovirinae* examples of Cherax quadricarinatus densovirus (CqDV) now renamed *Decapod aquambidensovirus 1* [previously Putative gill parvovirus] (genus *Aquambidensovirus*) in freshwater crayfish (*Cherax quadricarinatus*) in Queensland, Australia [9,10], sea star-associated densovirus in sunflower sea star (*Pycnopodia helianthoides*) on the Northeastern Pacific Coast [11], and Clinch densovirus 1 in freshwater mussel (*Acrimonies pectorosa*) in the Clinch River, Virginia, and Tennessee, USA [12].

*Hamaparvovirinae* members include hepatopancreatic parvovirus (HPV), now renamed *Decapod hepanhamaparvovirus 1* (DHPV-1) in the genus *Hepanhamaparvovirus* [formerly genus *Hepandensovirus*]; infectious hypodermal and hematopoietic necrosis virus (IHHNV), now renamed *Decapod penstylhamaparvovirus 1* in genus *Penstylhamaparvovirus* [formerly genus *Penstyldensovirus*] of prawns and shrimp; and in the genus *Ichthamaparvovirus*, *Syngnathid ichthamaparvovirus 1* of gulf pipefish (*Syngnathus scovelli*) and tilapia parvovirus (TiPV) of Nile tilapia (*Oreochromis niloticus*). Hepanhamaparvoviruses are widespread and highly pathogenic, causing hepatopancreatic disease, and constitute an economic threat in cultured shrimp populations when larvae from wild-caught shrimp are introduced [13] or virus-infected shrimp are used as live or fresh broodstock food or bait [14]. Penstylhamaparvoviruses were first identified in the 1980s in Hawaii as pathogens responsible for an economically significant and virulent disease in farmed shrimp (infectious hypodermal and hematopoietic necrosis) [15,16]; they are widespread but are no longer a major economic problem because of the development of domesticated genetically resistant specific pathogen-free (SPF) postlarvae shrimp [17,18,19,20,21,22,23]. However, IHHNV is still responsible for major economic losses on *Penaeus monodon* (Black Tiger shrimp) farms in countries like Australia, where it is endemic to *P. monodon* [24]. Ichthamaparvoviruses are parvoviruses of finfish, including tilapia parvovirus (TiPV), a recently identified viral infection associated with mass morbidity and mortality in farmed adult Nile tilapia in China [25] and farmed juvenile red hybrid tilapia in Thailand [26,27] and India [28]. The novel fish parvovirus (tilapia parvovirus HMU-HKU-1) was first identified using next-generation sequencing (NGS) on fecal samples from crocodiles fed with tilapia [29].

*Metalloincertoparvovirus* is an unassigned genus with one species, *Metalloincertoparvovirus decapod 1*, with the virus *Penaeus monodon* metallodensovirus (PmMDV) found in Vietnamese *P. monodon* [30,31].

### 1.1. Parvovirus Classification and Virion Properties

**Figure 1:**
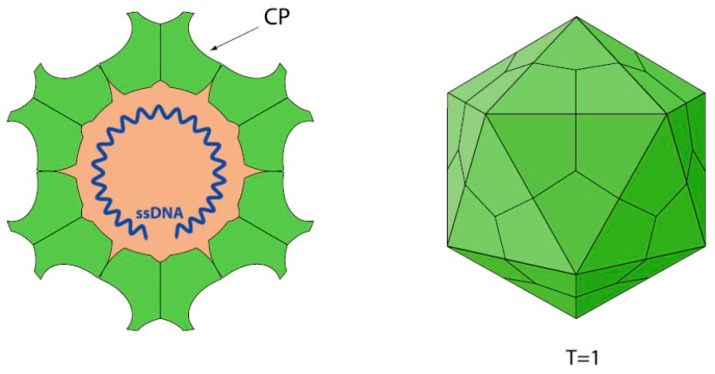
*Parvoviridae*. Schematic representation of a parvovirus particle showing the non-enveloped, round, T = 1 icosahedral symmetry, 22–28 nm in diameter. The capsid consists of 60 copies of CP protein. (Reproduced from [33]. Source: SwissBioPics. The images are licensed under a Creative Commons Attribution 4.0 International (CC BY 4.0) License https://creativecommons.org/licenses/by/4.0/ (accessed 21 December 2023)).

Parvoviruses are one of the smallest animal viruses known. “Parvus” is the Latin word for “small.” The virus particles are non-enveloped, T = 1 icosahedra, and 22–28 nm in diameter (Figure 1). They are extremely resistant to environmental conditions (pH 3–9; and 60 °C for 1 h) and survive for long periods outside the host cell, resulting in persistence in the environment, carriage on fomites, and wide dissemination [32]. The most reliable disinfection for terrestrial parvoviruses is achieved with common household bleach (3-6% sodium hypochlorite). Most vertebrate parvoviruses hemagglutinate red blood cells.

**Figure 2:**
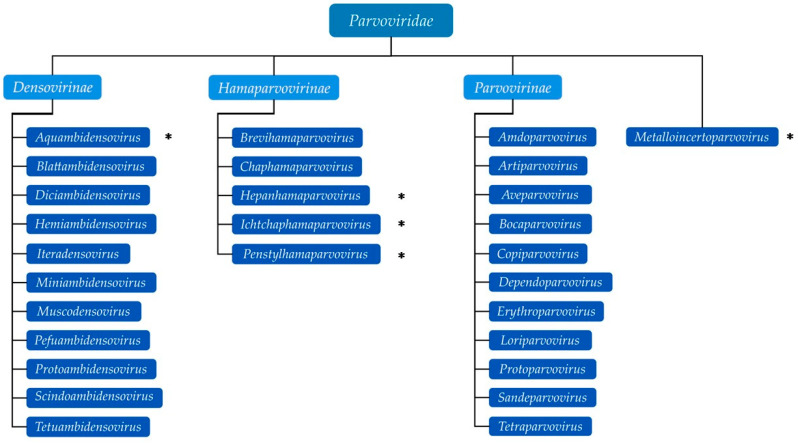
The current genera are listed by subfamily in the family *Parvoviridae*. A star indicates genera affecting marine animal hosts.

**Figure 3:**
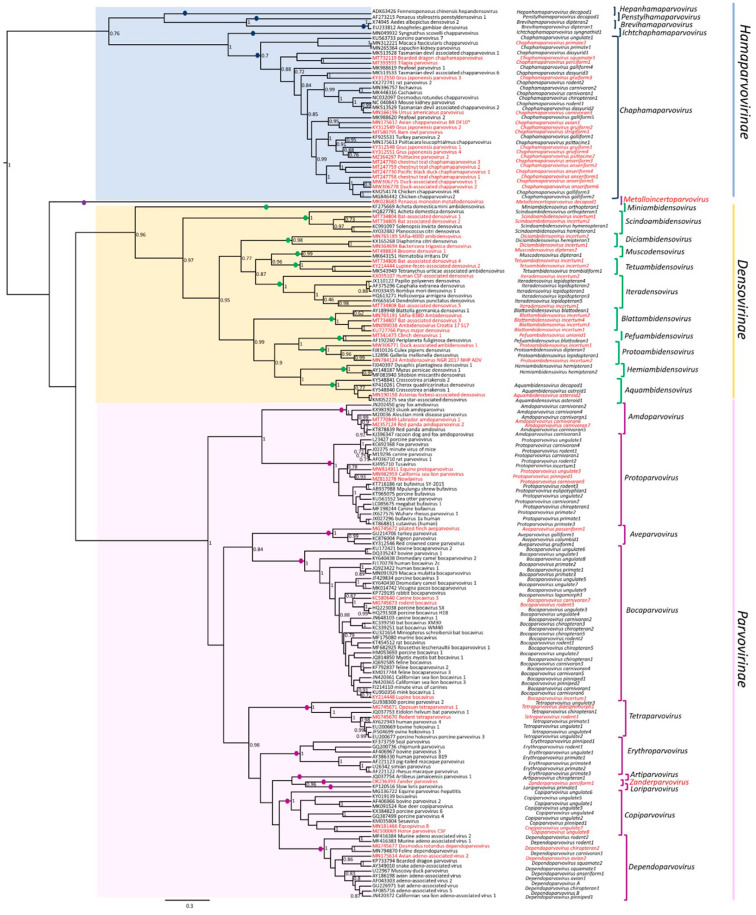
Bayesian inference of the entire *Parvoviridae* family, based on the SF3 helicase domain (155-aa-long) of the NS1 protein (by the BEAST v.1.10 suite using the LG+I+G substitution model, with a lognormal relaxed clock and Yule speciation model through 100 million generations). The posterior probability values are shown as node labels, if significant (>0.7). The nodes of each genus are labeled with a spot, color coded based on subfamily affiliation. Taxa and viruses introduced in this proposal are highlighted in red. Proposed taxa and viruses in the TP by Duarte et al. (create 1 new species in the genus *Chaphamaparvovirus*, and 1 new species in the genus *Dependoparvovirus*, in the family *Parvoviridae*) are marked with a star (*). (Reproduced from [31] with permission from Dr. Judit J. Pénzes, Chair, ICTV *Parvoviridae* Study Group.).

The genome is linear, monopartite ssDNA of a positive sense or negative sense (or ambisense in subfamily *Densovirinae*) of 3.9 to 6.3 kilobases. A divergent parvovirus *Acheta domesticus segmented densovirus* (AdSDV), genus *Brevihamaparvovirus 1*, subfamily *Densovirinae*, possesses a bipartite genome that is packaged as one genome segment per particle [34,35]. The *Parvoviridae* genome encodes a large non-structural protein (NS1) with a helicase superfamily 3 (SF3) domain and the capsid protein (VP) on one genome, whereas in AdSDV with a bipartite genome, the non-structural (NS) and structural (VP) protein expression cassettes are on two separate genome segments [34]. Parvoviruses are classified in the same species if their NS1 proteins share more than 85% amino acid sequence identity while diverging greater than 15% from members of other genera [1]. Two parvoviruses can be potentially classified in one genus if they cluster together on phylogenetic analysis of their complete NS1 protein sequences and SF3 helicase domains [1]. Additionally, their NS1 proteins should share 35–40% protein sequence identity and display at least 80% coverage between two members of the genus in question, but these numbers may be flexible [34]. Thus, using these NS1-based criteria, the family *Parvoviridae* is subdivided into three subfamilies, *Parvovirinae* with eleven genera that infect vertebrate hosts, *Densovirinae* with eleven genera that infect invertebrate hosts, and *Hamaparvovirinae* with five genera that infect both vertebrate and invertebrate hosts. The genus *Metalloincertoparvovirus*, which contains PmMDV that infects *Penaeus monodon* in Vietnam, is not assigned to a subfamily [31]. Figure 2 lists the genera of each subfamily indicating those affecting marine animal hosts to be elaborated on later. The evolutionary relationships of the different genera in the family *Parvoviridae* are shown in the phylogenetic tree in Figure 3. At the megataxonomic level, the family *Parvoviridae* has been classified in the realm Monodnaviria, kingdom Shotokuvirae, phylum Cossaviricota, class Quintoviricetes, and order Piccovirales, based on its relations to other DNA virus taxa [1].

#### 1.1.1. Parvovirus Genome Organization

**Figure 4:**
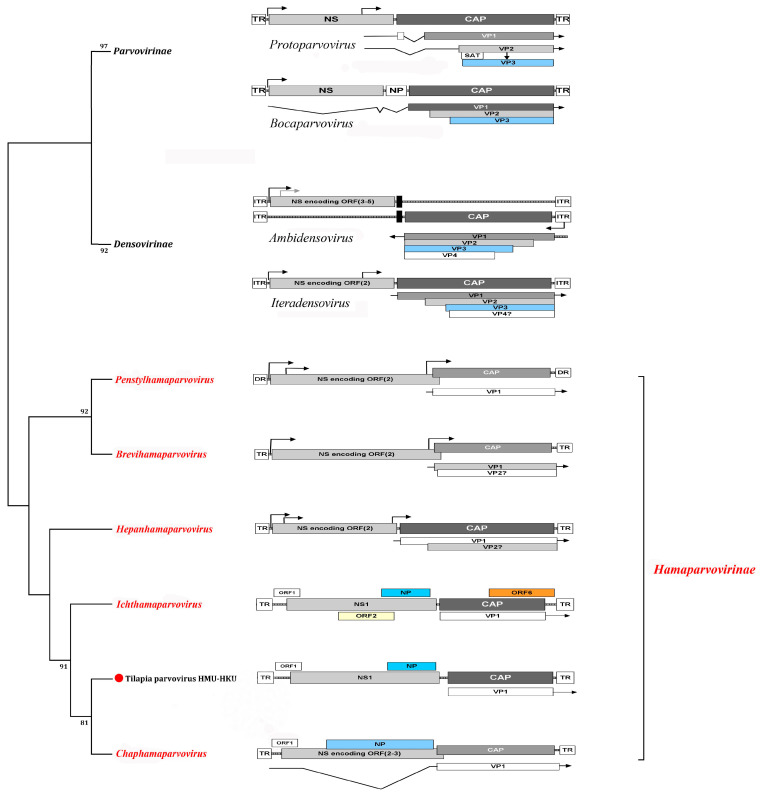
Cladogram of subfamilies *Hamaparvovirinae*, *Parvovirinae*, and *Densovirinae*. The genome organizations of members of the representative genera of the three subfamilies are shown. The novel tilapia parvovirus HMU-HKU-1 discovered in this study is labeled with a red circle (●). The genera and subfamilies described in the newly proposed ICTV parvovirus taxonomic classification are highlighted in red. (Reproduced under Creative Commons Attribution License (https://creativecommons.org/licenses/by/4.0/ (accessed 21 December 2023)) from [29], Figure 2).

The coding region in the genome is flanked by two inverted terminal repeat (ITR)-containing palindromic double-stranded hairpin ends required for replication. Still, many other characteristics vary between members of different genera. Figure 4 shows the genome organizations of members of the representative genera of the three subfamilies recognized by the International Committee for the Taxonomy of Viruses (ICTV), *Parvovirinae*, *Densovirinae*, and *Hamaparvovirinae* [29]. All parvoviruses encode the non-structural (NS) protein gene(s) and genes of accessory proteins in a negative-sense orientation on the left-hand side of the genome. In contrast, the structural capsid VP gene on the right-hand side may be in a negative-sense orientation (subfamilies *Parvovirinae* and *Hamaparvovirinae*) or positive-sense orientation (i.e., ambisense genome in subfamily *Densovirinae*). All parvovirus genome 3′- and 5′-termini contain ITRs that form self-priming palindromic hairpin telomeres which function as viral DNA replication origins [36]. These secondary structures can either be the same or different at the 5′- and 3′-termini, leading to homotelomeric or heterotelomeric genomes, respectively, and are consistent across a genus. Among the subfamilies *Parvovirinae* and *Hamaparvovirinae*, homotelomeric viruses package equal numbers of plus or minus stranded genomes in viral particles. A packaging bias toward one viral genome strand is observed in parvoviruses with heterotelomeric genomes. For example, some parvoviruses encapsidate only the negative-sense DNA strand (e.g., *Carnivore protoparvovirus 1*, such as Canine parvovirus, and genus *Amdoparvovirus*) [37]; others encapsidate different portions of either positive-sense or negative-sense DNA strands (e.g., *Primate erythroparvovirus 1* or Human parvovirus B19) and members of genus *Dependoparvovirus* package both strands in separate particles in a ratio of 1:1. Many parvoviruses package predominantly negative DNA strands. The proportion of negative to positive strands packaged depends on the host cell infected. No parvovirus is known to package predominantly positive DNA strands; positive-strand DNA occurs in variable proportions of 1–50% (so the genome of parvoviruses is often described as ssDNA of a negative sense). The NS gene forms one or more nonstructural proteins (NS1-NS3) via alternative mRNA splicing [23]. Parvovirus NS1 is a large multifunctional protein with strand- and site-specific endonuclease (nicking) activity, ATPase activity, and an SF3 helicase domain with 3′ to 5′ processivity, rolling circle replication initiator protein motifs, and DNA binding domains; it is absolutely required for virus replication [38] and also contributes to pathology [39]. Parvovirus capsids can compose up to four VPs (VP1-4) generated from a single ORF of the VP gene by alternative splicing, and all share a large C-terminal region [38].

#### 1.1.2. Virus Replication

**Figure 5:**
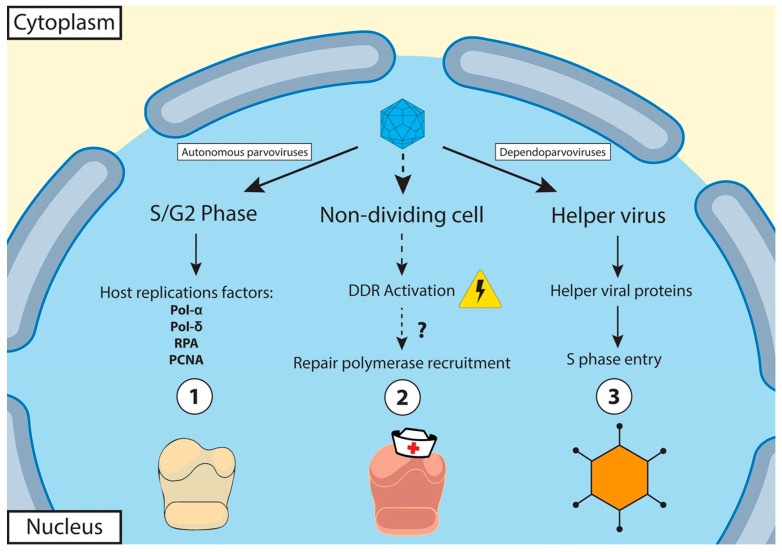
Summary of parvovirus replication requirements. (1) Most autonomous parvoviruses require mitotically active cells (S/G2 phase) to provide host replication factors to replicate their viral genome. (2) Recently, human bocavirus 1 (HBoV1) was demonstrated to replicate in non-dividing airway epithelial cells through the hijacking of DNA repair machinery [42,43]. (3) Dependoparvoviruses depend on coinfection with a helper virus to undergo productive replication in a host cell. (Reproduced under Creative Commons Attribution License (https://creativecommons.org/licenses/by/4.0/ (accessed 21 December 2023)) from [38], Figure 4).

Parvoviruses replicate in the nucleus, producing large intranuclear inclusion bodies. Parvoviruses enter the cell by receptor-mediated endocytosis and replicate their genome in the nucleus by “rolling hairpin replication”, a unidirectional single-strand displacement mechanism, using the viral self-priming hairpin telomeres as well as cellular polymerases, ligases, and other replication factors [40]. In many parvoviruses, the extended N-terminus of the VP1 protein includes the phospholipase A2 (PLA2) enzymatic domain that is essential for cell entry due to the need to release from the endosomal or lysosomal pathway [36,41]. The crustacean parvoviruses that infect penaeid shrimps belonging to the genera *Hepanhamaparvovirus* (HPV) and *Penstylhamaparvovirus* (IHHNV), subfamily *Hamaparvovirinae*, and the undefined genus *Metalloincertoparvovirus* (PmMDV) lack the PLA2 domain and therefore have a different mechanism for endosomal/lysosomal egress [1,30,38]. Because of their small genome, parvoviruses lack a viral DNA polymerase and require actively dividing host cells, although they cannot induce cells into the S phase (Figure 5). Members of the genus *Dependoparvovirus* are defective and require a helper adenovirus or herpesvirus for replication.

### 1.2. General Pathogenesis of Parvoviruses

In general, the disease outcome of a parvovirus infection is controlled by various factors [38]. For example, in the autonomous parvoviruses, virus replication is dependent on cellular functions provided in the S phase of the cell division cycle, i.e., when cellular DNA synthesis is occurring. This feature is correlated with the pathogenic potential of these viruses, so tissues with high cellular division rates are disproportionately affected [38]. Among the parvoviruses identified in aquatic animals, *Cherax quadricarinatus* densovirus (CqDV), *Cherax destructor* systemic parvo-like virus (CdSPV) [9,10], and Clinch densovirus 1 [12] in the subfamily *Densovirinae* are pathogenic. In contrast, other densoviruses of wild aquatic animals are not consistently associated with disease [11,44]. Hepatopancreatic parvovirus (HPV) [45], infectious hypodermal and hematopoietic necrosis virus (IHHNV) [46], and tilapia parvovirus (TiPV) [25] in the subfamily *Hamaparvovirinae* are highly pathogenic in both wild and farmed aquatic animal species. As would be expected, most of the viruses discovered through metagenomic sequencing of the aquatic animal host virome, such as Zander parvovirus [5], salmon parvovirus [6], and *Crangon crangon* parvo-like virus 1 (CcPaLV 1) [7] in the subfamily *Parvovirinae,* cause little or no disease.

The risk factors for potentially pathogenic parvoviruses causing disease in infected aquatic animals include host factors such as age (neonates and juveniles are usually more susceptible), immune status of the host (immune system, cytokines, etc.), usually exacerbated by stress due to aquaculture activities such as handling and stocking density, and host species. Immunosuppressed animals are frequently affected by coinfections which precipitate overt disease. With regards to coinfections, it should be noted that the requirement for cycling cells, thereby targeting tissues with a high cell turnover (or rapidly dividing cells) by autonomous parvoviruses, may explain the occurrence of parvoviruses in multiple-type infections (i.e., mixed infections or coinfections) in non-immune tissues, in addition to those parvoviruses that cause immunosuppression because the immune tissues characteristically have high cell turnovers. Shrimp viruses can spread horizontally through cannibalism among shrimp, which is facilitated by high stocking densities [47]. Non-host risk factors that might exacerbate disease severity in both wild and farmed aquatic animals susceptible to parvovirus infection are environmental factors, such as oxygen, salinity, temperature, pH, water flow, etc., which are also important in terms of the survival of the virus and its transmission in the environment. For example, virus-infected shrimp are used as live or fresh broodstock food or bait, facilitating virus spread between farms and countries [14]. Water, habitat (crab), and live food (squid, krill, and polychaeta) were suspected to be sources of DHPV-1 in shrimp hatcheries [14]. Densoviruses contaminating the habitat have been associated with mass mortalities in echinoderms [11] and freshwater mussels [12]. Environmental stressors may also affect the clinical outcome of parvovirus-infected sea stars [48,49]. 

**Figure 6:**
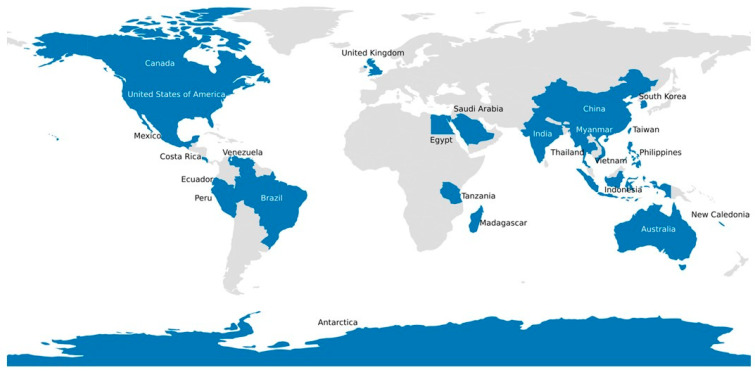
The geographic origin of *Parvoviridae* sequences from marine animal hosts. The *Parvoviridae* sequences were downloaded from the NCBI Virus Sequences for Discovery portal [64].

Table 1 provides details of parvoviruses found in different aquatic animal species with subfamilies and genera of the respective parvovirus species. A map of the geographic origin of *Parvoviridae* sequences from marine animal hosts is shown in Figure 6. The genetic basis of classification into the three different subfamilies has been described in Section 1.1.1. Members of the subfamily *Densovirinae* infect only invertebrates, including *Decapod aquambidensovirus 1* of crayfish, *Asteroid aquambidensovirus 1* of sea stars and sea urchins, and Clinch densovirus 1 of freshwater mussels, characteristically causing mass mortality in their aquatic animal hosts with common signs including lethargy, anorexia, flaccidity, and death [12]. Members of the subfamily *Hamaparvovirinae* infect both invertebrates and vertebrates; *Decapod hepanhamaparvovirus 1* and *Decapod penstylhamaparvovirus 1* of prawn and shrimps were also discovered through investigations of mass die-offs in their aquatic animal hosts, whereas tilapia parvovirus of Nile tilapia, although first identified using next-generation sequencing on fecal samples from crocodiles fed with tilapia [29], has also been shown to cause mass mortality of farmed tilapia [25]. These aquatic hamaparvoviruses commonly occur in multiple infections with other more pathogenic agents [26,27,50,51] and may cause asymptomatic infection in adult carrier hosts [52] or become endogenous viral element (EVE) sequences in the host genome following chronic or persistent infection [53]. Members of the subfamily *Parvovirinae* also infect both invertebrates and vertebrates: Zander parvovirus of zander or pikeperch [5], salmon parvovirus of sockeye salmon smolts [6], and *Crangon crangon* parvo-like virus 1 of mollusks [7] were all discovered by using viral metagenomics, and their pathogenic potential remains unknown.

**Table 1:** Summary of parvoviruses found in aquatic animals.

Subfamily and Genus	Species ^1^	Virus Common Name (Abbreviation)	Sampled Host	Geographical Area ^2^	Clinical Disease and Common Clinical Signs	Reference
Densovirinae, Aquambidensovirus	*Decapod aquambidensovirus 1*	Cherax quadricarinatus densovirus [previously known as Cherax quadricarinatus parvo-like virus (CqPlV)]; Cherax destructor systemic parvo-like virus (CdSPV)	redclaw crayfish Cherax quadricarinatus and Cherax destructor	Queensland, Australia	Decrease in stress resistance manifested as chronic mortality or mass mortality of up to 96% cumulative mortality. In experimental infection, inoculated crayfish showed gross signs of malaise, anorexia, and disorientation before dying. Tissues of endodermal, ectodermal, and mesodermal origin have enlarged nuclei containing intranuclear inclusions. Transmission electron microscopy showed the inclusions to consist of viral particles 19.5 nm in diameter.	[9,10]
	*Asteroid aquambidensovirus 1*	Sea star-associated densovirus (SSaDV)	sea stars and sea urchins	Pacific coast of USA and Atlantic coast of North America	Originally associated with sea-star wasting disease (SSWD) causing mass mortality in asteroids. Affected animals show behavioral changes, various lesions such as white lesions along the arms progressing to large areas of exposed underlying white ossicles, loss of turgor, limb autonomy (“dropping” of the limbs), and death characterized by rapid degradation (“melting”). Histologically, pink round to irregular inclusions were noted in the vacuolated nuclei of affected epithelial cells in some samples.	[11,54]
		Clinch densovirus 1	Freshwater mussels	Clinch River, Virginia, and Tennessee, USA	Episodic mass mortality with moribund mussels appearing on the surface of the substrate, gaping, slow to respond to tactile stimuli, and able to close their valves only weakly. Histologic lesions consisted of pervasive necrosis of the internal organs.	[12]
Hamaparvovirinae, Hepanhamaparvovirus	*Decapod hepanhamaparvovirus 1*	Fenneropenaeus chinensis hepatopancreatic densovirus [previously known as Hepatopancreatic parvovirus (HPV)]	Prawns and Shrimp	Australia, Brazil, China, El Salvador, India, Indonesia, Israel, Kenya, Madagascar, Malaysia, Mexico, New Caledonia, Philippines, Singapore, South Korea, Taiwan, Tanzania, Thailand, and the United States of America	Reduced growth in juvenile shrimp, anorexia, reduced preening with a concurrent increase in surface, and gill fouling by epicommensal organisms. Increased mortalities during the larval stages with signs of necrosis and atrophy of the hepatopancreas. Hepatopancreatic cells and epigastric caecal epithelial cells have hypertrophied nuclei with basophilic inclusion bodies. Transmission electron microscopy showed the intranuclear inclusion bodies to consist of viral particles 22–24 nm in diameter.	[45,50,55,56,57]
Hamaparvovirinae, Ichtahamaparvovirus	*Syngnathid ichthamaparvovirus 1*	Syngnathus scovelli chapparvovirus	gulf pipefish	Gulf of Mexico and parts of South America	Not known.	[58]
		Tilapia parvovirus (TiPV)	Nile tilapia	China, India, and Thailand	Mass morbidity and 60–70% mortality in adult Nile tilapia (500–600 g); mass mortality of 50–75% in juvenile red hybrid tilapia (10–30 g to 300–800 g). Clinical signs in affected fish include lethargy, reduced feeding, scale loss, redness on body with hemorrhages on the operculum, base of fins and ventral part, opaqueness of eyes, swimming near the pond edge or abnormal swimming patterns before death. TiPV targets the pancreas and the presence of Cowdry type A inclusion bodies in acinar cells of the pancreas is pathognomonic of TiPV infection	[25,26,27,28]
		unclassified Ichthamaparvovirus				
Hamaparvovirinae, Penstylhamaparvovirus	*Decapod penstylhamaparvovirus 1*	Penaeus stylirostris penstyldensovirus (PstDV 1&2); Penaeus monodon penstyldensovirus (PmoPDV 1&2) [previously known as infectious hypodermal and hematopoietic necrosis virus (IHHNV)]	Prawns and Shrimp	Australia, Brazil, Caribbean, Central America, Ecuador, Indonesia, Israel, Malaysia, New Caledonia, Peru, Philippines, Singapore, Tahiti, Taiwan, Thailand, and United States of America	IHHN; 80–100% mortality in postlarvae and juveniles of Penaeus (Litopenaeus) stylirostris and postlarvae of *Macrobrachium rosenbergii*; runt deformity syndrome (RDS) in juvenile of *P. vannamei* and *P. monodon*; >80% mortality in postlarvae and chronic mortality in juveniles of *Penaeus esculentus* X *Penaeus monodon* hybrids. The animals become weak and stop moving prior to death, and the gills have many small melanized foci. There are white or buff colored spots in the cuticular epidermis, especially at the junction of the tergal plates of the abdomen, giving the shrimp a mottled appearance. Histologically, in acute infections, the gills, lymphoid organ, other organs, and connective tissue show intranuclear Cowdrey’s type A inclusions in hypertrophied nuclei. Transmission electron microscopy showed the inclusions to consist of viral particles 17–26 nm in diameter.	[46,59,60,61,62]
Unassigned genus	*Metalloincertoparvovirus decapod 1*	*Penaeus monodon* metallodensovirus (PmMDV)	Vietnamese *P. monodon*	Vietnam	Mass mortality in *P. monodon*. Clinical signs include a red telson, uropodia, and pleopods, and discoloration of the cephalothorax.	[30]
Unassigned		Novel salmon parvovirus	Sockeye salmon	BC, Canada	Not known	[6]
Unassigned in subfamily Parvovirinae		Novel zander parvovirus (zander/M5/2015/HUN, OK236393)	Zander or pikeperch (Sander lucioperca)	Hungary	Not known	[5]
Unassigned		Crangon crangon parvo-like virus 1 (CcPaLV 1)	Molluscs	Europe	Not known	[7]

^1^ Species as listed in the NCBI Taxonomy Browser [63]. ^2^ See Figure 6 for the map.

### 1.3. Challenges Related to Controlling Parvoviruses in Aquatic Animals

Similarly to terrestrial animal parvoviruses, aquatic animal parvoviruses are resistant to environmental conditions and persist in the environment, making the control of parvovirus diseases difficult. For various reasons, there are currently no vaccines against aquatic animal parvoviruses. The differences in the immune system of aquatic animals compared to terrestrial animals [65,66] dictate that vaccination is not as effective in controlling aquatic animal parvoviruses as it is for terrestrial animal parvoviruses; in fact, it is not an option in crustaceans and mollusks. Prophylactic measures to reduce parvovirus infection in farmed aquatic animals (tilapia and shrimps) include feeding probiotic and prebiotic supplements to enhance animal health and to modulate the animal’s gut microbiota to improve gut health, and herbal medicines and immunostimulants to enhance immunity to disease [67,68]. The best strategy to control these viruses includes improved farm management and biosecurity methods involving prevention, control, and eradication of the viruses from the farms, including the disinfection of ponds, filtration of water, and treatment of effluents, in addition to monitoring the fluctuations of abiotic factors, such as oxygen, salinity, and temperature. An important aspect of biosecurity is implementing genomic surveillance of viral pathogens and developing and standardizing diagnostic techniques. It is also crucial to characterize the pathology presentations of the viral diseases, especially since many of the aquatic animal parvoviruses are emerging in aquatic hosts, and there is a lack of knowledge with regards to the farmed species that are important for aquaculture.

Tilapia is the world’s most extensively farmed species after carp, with global production reaching USD 12.3 billion in 2020 with a global commodity trade (export, import, and re-export) value of USD 3.2 billion [69] and it is expected to reach USD 9.2 billion by 2027 [70]. Although there are efficacious commercially available vaccines used in tilapia aquaculture [71], none are available for use against tilapia parvovirus (TiPV) infection. This is because TiPV is a recently emerging pathogen in tilapia aquaculture and biological studies are still in their infancy. However, fish vaccine development and availability is one of the major issues in aquaculture, in addition to biosecurity protocols, genomic surveillance of viral pathogens, and advances in diagnostics. Implementing disease-free stocks is a big concern when it comes to invertebrates. In shrimp aquaculture, where vaccination is not an option, biosecurity, including hatchery-based PCR screening of individual broodstock and pools of postlarvae to ensure that only virus-free or virus-low seedstock are farmed, and the development of domesticated genetically resistant specific pathogen-free (SPF) postlarvae shrimp [17,18,19,20] remain the cornerstone in managing viral diseases [21,22,23,24]. Gene silencing using the RNA interference (RNAi) approach has been reported for both HPV [72,73] and IHHNV via injection. However, the oral delivery of RNAi molecules remains a challenge, and the utility of RNAi-based therapy has yet to be materialized in shrimp aquaculture [21,68]. A potential application of RNAi is in treating infected larvae or mature female broodstocks to generate virus-free or virus-low shrimp progeny. However, issues of safety and public acceptance of a genetic engineering technique must be considered for future application in field trials. Indeed, although immunostimulants, vaccines, and RNAi provide promising results in laboratory studies, the use of all these methods in the field may not be reliable, especially because of limitations in their efficacy, practicality, cost, and reproducibility [68]. 

The viruses that pose the greatest threat in aquaculture and challenge the sustainability and productivity of this industry are listed/reportable to the World Organization for Animal Health (WOAH) [74]. While mortality rates due to TiPV can reach 100% [28], the virus, which was first reported in 2019, has not yet been listed by the WOAH. Aquatic viral pathogens account for most of the 40% production loss (~USD 3 billion annually) attributed to disease in tropical shrimp production [75]. The WOAH list of reportable diseases currently includes only one aquatic animal parvovirus, *Penaeus stylirostris penstyldensovirus 1* (PstDV1) (formerly infectious hypodermal and hematopoietic necrosis virus, IHHNV) [74]. IHHNV disease outbreaks in the early 1980s led to the collapse of penaeid shrimp farming in the Americas [76], but the importance of this virus has significantly declined with the introduction of domesticated highly tolerant SPF stocks of *Penaeus* (*Litopenaeus*) *vannamei* (White Pacific shrimp) in shrimp aquaculture [77]. However, the virus is still responsible for major economic losses in *Penaeus monodon* (Black Tiger shrimp). IHHNV affects different shrimp species in three ways, depending on the host species and virus genotype. In unselected *Penaeus stylirostris*, IHHNV causes an acute, often catastrophic disease with mortality rates approaching 100%. In contrast to *P. vannamei*, some selected lines of *P. stylirostris* and *P. monodon* experience a more subtle, chronic disease known as runt deformity syndrome (RDS), characterized by significant growth suppression and cuticular deformities but not typically high mortality. Additionally, some genetic lines of *P. monodon* have been found to contain endogenous viral element (EVE) sequences [60,78]. According to Sellars et al. [24], *P. monodon* farms that stocked postlarvae shrimp with high levels of IHHNV infection experienced diminished performance, decreased survival rates, increased food conversion ratios, and production losses estimated at approximately AUD 67,000 per hectare of farm gate value. In contrast, farms that stocked postlarvae with low-to-negligible IHHNV viral loads exhibited significantly better outcomes [24]. IHHNV and *Decapod hepanhamaparvovirus 1* (DPHV-1) (formerly Hepatopancreatic parvovirus, HPV) were spread widely, likely unknowingly, as the shrimp aquaculture industry grew massively in the early 1980s before molecular detection methods were available. HPV was removed from the WOAH list as it is no longer associated with any significant negative economic repercussions in the aquaculture industry [77]. However, this virus remains in circulation in penaeid shrimp in several countries [77,79,80,81,82,83] and can cause considerable losses in shrimp grow-out ponds without any obvious clinical manifestation [21]. For example, a screening of *P. monodon* postlarvae from aquaculture hatcheries in India detected HPV and IHHNV alone or in combination in 93.3% of the samples; this very high rate of prevalence of these viruses is attributed primarily to a lack of screening strategies for the presence of these viruses in India [82]. In South Korea, DHPV-1 has been detected in samples from pond water, shrimp, feces, feed, crabs, and barnacles [83]. Moreover, DPHV-1 infection is seldom observed alone in epizootics and has occurred in multiple infections with other more pathogenic agents [14,50,51,80,81,84,85,86], making it a significant threat to shrimp aquaculture [14]. In Australia, IHHNV is endemic in *P. monodon* and has been detected at a high prevalence in farmed shrimp [24]. For the control of IHHNV and HPV, it is necessary to monitor the shrimp themselves and live food (such as squid, krill, polychaeta, and artemia) that is fed to the shrimp throughout all stages of production, including broodstock and postlarvae, and it is equally important to monitor and manage habitats surrounding the farms to protect shrimp from disease [14,24]. How many viral species in the *Parvoviridae* family will affect aquaculture economically is still unknown.

## 2. Genus Aquambidensovirus

The term “Ambidensovirus” was coined in 2014 as the name of a genus in the family *Parvoviridae* containing members with an ambisense genome organization [87]. *Aquambidensovirus* contains those viruses known to infect aquatic animal hosts, which currently include two assigned species, *Decapod aquambidensovirus 1* and *Asteroid aquambidensovirus 1*. Members of the genus share about 70% NS1 amino acid sequence identity and ~30% with other members of the subfamily *Densovirinae* [1]. A novel densovirus, Clinch densovirus 1, had a 63% amino acid sequence identity with *Periplaneta fuliginosa densovirus* (AF192260), which infects smoky brown cockroaches (*Periplaneta fuliginosa*) in China, the closest phylogenetic relative in the GenBank database [12], and did not cluster with *Asteroid aquambidensovirus 1* [1], suggesting that it is a different species, possibly the third species in genus *Aquambidensovirus*.

### 2.1. Decapod Aquambidensovirus 1 (Cherax Quadricarinatus Densovirus (CqDV))

*Decapod aquambidensovirus 1* is the type species of the genus *Aquambidensovirus*, with only one virus, *Cherax quadricarinatus* densovirus (CqDV) [previously Putative gill parvovirus] [9]. The virus was first identified in a single moribund freshwater crayfish (*Cherax destructor*) collected in South Australia [88]. It was subsequently identified in a redclaw crayfish (*Cherax quadricarinatus*) farm with chronic mortalities in mixed infection with presumptive hepatopancreatic reovirus [9] and in another commercial redclaw crayfish farm with mass mortality in northern Queensland, Australia [10]. The main histopathological lesion in *Cherax quadricarinatus* in the transmission trial conducted by Edgerton et al. [9] was hypertrophic gill nuclei. The mass mortality reported by Bowater et al. [10] occurred in juvenile animals over four weeks (96% cumulative mortality); affected crayfish were weak, anorexic, and lethargic. Experimentally inoculated crayfish showed gross signs of malaise, anorexia, and disorientation before dying, and the gills, cuticular epithelium, and epithelial cells of the foregut, midgut, and hindgut were the most heavily infected tissues; virions consistent with the parvovirus morphology were seen by electron microscopy in the enlarged nuclei of both naturally and experimentally infected crayfish [10]. In the infection trial of redclaw crayfish infected with CqDV conducted by Bochow [89], the clinical signs appeared from 17 to 57 days post-injection. The most obvious lesion consisted of blisters filled with a viscous, gelatinous substance on the inner membrane between the carapace and gills extending across the branchial cavity covering the top underside of the cephalothorax (shown in Figure 4.1 of Bochow [89]). Microscopically, large basophilic intranuclear inclusion bodies, characteristic of parvovirus infection, were present in ectodermal tissue cells of the gills, cuticular epithelium, and gastric sieve and hemocytes in the hemal spaces (shown in Figure 4.2 of Bochow [89]).

CqDV has one of the largest genomes in the family *Parvoviridae* (6334 nucleotides) (GenBank Accession # KP410261) [90]. Bochow [89] reported primers for the molecular detection of CqDV by qPCR with SYBR Green detection (Table 2). Analysis of the tissue tropism of CqDV in six organs using this qPCR method showed that the branchial epithelium had the highest mean copy number mg-1 of tissue (1.22 × 10^6^), followed by the pleopod (1.19 × 10^6^), antennal gland (5.53 × 10^5^), gill (4.66 × 10^5^), heart (1.32 × 10^5^), and muscle (6.12 × 10^4^) [89].

### 2.2. Asteroid Aquambidensovirus 1 (Sea Star-Associated Densovirus (SSaDV))

The species *Asteroid aquambidensovirus 1* comprises three viruses identified in sea stars and sea urchins (phylum Echinodermata) [11,102], and all are highly pathogenic [1]. Sea star-associated densovirus (SSaDV), the only one classified to date, was associated with an extensive outbreak of sea-star (asteroid) wasting disease (SSWD) (also known as “asteroid idiopathic wasting syndrome”) with mass mortality of captive asteroids in three species of common asteroid (*Pycnopodia helianthoides*, *Pisaster ochraceus*, and *Evasterias troschelii*) on the Northeastern Pacific Coast in 2013–2014 [11]. Environmental stressors may also play a role in the clinical outcome of infected sea stars [48,49], as a reexamination of the original metagenomic data found the virus commonly associated with apparently healthy or asymptomatic animals [91]. Clinical signs of SSWD include abnormal twisting of appendages, followed by the formation of white lesions and loss of turgor or a “deflated” appearance (deflation of arms and body), progressing to arm loss, necrosis, and rapid degradation leading to death [11,44].

Jackson et al. [91] reported primers for molecular detection of SSaDV by conventional PCR targeting VP1 (Table 2). When this PCR assay was used to assess putative tissue tropism in three sea star species (*Pisaster ochraceus*, *Evasterias troschelii*, and *Pisaster brevispinus*), SSaDV was detected most frequently in the pyloric caeca (40/45 or 89%), followed by the tube feet (17/47 or 36%), stomach (5/46 or 11%), body wall (5/47 or 11%), and gonads (4/42 or 10%) [91].

### 2.3. Clinch Densovirus 1

Clinch densovirus 1 is a novel densovirus that has been linked to mass mortality in freshwater mussels, pheasantshell (*Acrimonies pectorosa*), in the Clinch River, Virginia, and Tennessee, USA, since 2016 [12]. The virus was 1 of 17 novel viruses identified using hemolymph for metagenomic sequencing for virus discovery and the only one that was epidemiologically linked to morbidity. Clinch densovirus 1 is currently an unassigned species. Phylogenetic analysis showed that it did not cluster with *Asteroid aquambidensovirus 1* [1], suggesting that it is a different species, possibly the third species in the genus *Aquambidensovirus*.

## 3. Genus Hepanhamaparvovirus

*Hepanhamaparvovirus* is one of three genera (together with genera *Penstylhamaparvovirus* and *Ichthamaparvovirus*) containing viruses known to infect aquatic hosts in the new subfamily *Hamaparvovirinae* (Figure 2). Hepanhamaparvoviruses possess the largest genome, ∼6.3 kb, with 220 nt long ITRs that form hairpins (the *Ichthamaparvovirus* genome is ~4.3 kb also with ITRs that form hairpins, whereas the *Penstylhamaparvovirus* genome is only 3.9 kb long and instead of hairpins harbors direct terminal repeats) [103]. Members of the subfamily *Hamaparvovirinae* have an average of 30% amino acid sequence identity of their NS1 protein, and all species, like members of genera *Aveparvovirus* and *Amdoparvovirus* (subfamily *Parvovirinae*), lack the otherwise conserved phospholipase A2 domain in their VP1 proteins [1,38]. The genus *Hepanhamaparvovirus* has one species, *Decapod hepanhamaparvovirus 1* (DHPV-1), also the type species of its former genus, *Hepandensovirus*.

### Decapod Hepanhamaparvovirus 1 (DHPV-1) (Hepatopancreatic Parvovirus (HPV))

The species *Decapod hepanhamaparvovirus 1* (DHPV-1) was previously known as hepatopancreatic parvovirus (HPV), formerly in the genus *Hepandensovirus*, and is widely distributed in both wild and farmed shrimps worldwide, including Australia, Brazil, China, El Salvador, India, Indonesia, Israel, Kenya, Madagascar, Malaysia, Mexico, New Caledonia, Philippines, Singapore, South Korea, Taiwan, Tanzania, Thailand, and the United States of America. The hosts for this virus do not occur in Canada [45]. DHPV-1 includes several genetically distinct strains from different shrimp and prawn species in different countries [51,104,105,106]. The genomes of the following ten strains have been completely sequenced: *Penaeus monodon hepandensovirus 1* (PmoHDV1 (Thailand), GenBank Accession# DQ002873.1); *Penaeus chinensis hepandensovirus* (PchDV (China), Accession # NC_014357); *Penaeus monodon hepandensovirus 2* (PmoHDV2 (Madagascar), Accession #s EU247528.1 and MT980830); *Penaeus monodon hepandensovirus 3* (PmoHDV3 (Tanzania), Accession# EU588991.1); *Penaeus merguiensis hepandensovirus* (PmeDV (Australia), Accession# DQ458781.4); *Penaeus monodon hepandensovirus 4* (PmoHDV4 (India), Accession# FJ410797.2); and *Fenneropenaeus chinensis hepandensovirus* (FchDV (South Korea), Accession# JN082231.1; (China) Accession# GU371276.1; and (Korea) Accession# AY008257). Another strain of DHPV-1 that has been refractory to PCR methods designed for DHPV-1 detection in *P. monodon* (Black tiger shrimp) [106] occurs in cultivated giant river prawn *Macrobrachium rosenbergii* in Thailand [79,107] and Malaysia [108,109]. Lee et al. reported a novel genotype of DHPV-1 in *P. vannamei* (Pacific white shrimp) with approximately 70% sequence identity with all known DHPV-1s and with a unique ten amino acid deletion and 3 and 1 amino acid insertions in the VP gene in a mixed infection with *Enterocytozoon hepatopenaei* in Taiwan [80] and South Korea [83]. The novel DHPV genotype was also reported in coinfection with white spot syndrome virus (WSSV) in cultured *P. vannamei* in South Korea [14]. Thus, to date, at least four genotypes of DHPV-1 can be defined based on the VP gene sequence, as shown in Figure 4 of Lee et al. [80]. Genotype I consists of strains from South Korea, China, Madagascar, and Tanzania; Genotype II includes strains from India, Indonesia, and Thailand; Genotype III includes strains from Australia and New Caledonia; and Genotype IV is the novel strain from Taiwan and South Korea [14,80,83].

DHPV-1 is widespread and highly pathogenic, causing hepatopancreatic disease, and it can constitute an economic threat in cultured shrimp populations on rare occasions when larvae from wild-caught shrimp are introduced. HPV was first described from farmed *Penaeus merguiensis* and *P. indicus* with a mixed infection with chlamydia in Singapore [110] and is considered to have later spread to wild shrimp in the Americas via the importation of live infected Asian shrimp for aquaculture [111]. The host range of DHPV-1 includes at least 19 species of wild and cultured shrimp, prawns, and crabs worldwide [51]; it has been reported in many countries, including Australia, China, Korea, the Philippines, Indonesia, Malaysia, India, Kenya, Kuwait, Israel, and Taiwan, as well as from the Americas [51,80]. DPHV-1 was removed from the WOAH list of reportable pathogens as it is no longer associated with significant negative economic repercussions in the aquaculture industry [77]. However, the virus remains in circulation in penaeid shrimp in China [77], Thailand [79], India [81,88], Taiwan [80], South Korea [14,83], and several other countries, and it can cause considerable losses in shrimp grow-out ponds without any obvious clinical manifestation [22]. A novel strain of DPHV-1 that does not cause histopathology in *P. monodon* was recently discovered in Madagascar [23]. Moreover, DPHV-1 infection is seldom observed alone in epizootics and has occurred in multiple infections with other more pathogenic agents [50,51], which likely downplays its pathogenicity and economic significance. For example, there have been reports of coinfection of DHPV-1 and *Enterocytozoon hepatopenaei* (EHP) in *P. vannamei* [80,81]; DHPV-1 and WSSV in *P. vannamei* [14]; DHPV-1 and monodon baculovirus (MBV) in *P. monodon* [84,85]; DHPV-1 and WSSV in *P. monodon* [99]; triple infection with DHPV-1, MBV, and Yellow head virus (YHV) in *P. monodon* [86]; DHPV-1, MBV, and WSSV in *P. monodon* [50]; and quadruple infection with DHPV-1, WSSV, IHHNV, and MBV in *P. monodon* [112].

DHPV-1 infects the epithelial cells of the hepatopancreas and midgut of shrimp [97], with infected individuals showing non-specific gross signs, including an atrophied hepatopancreas, anorexia, retarded growth, and reduced preening activities, resulting in epifouling in gills and appendages [113]; most DPHV-1-infected juvenile shrimp simply grow very slowly, stopping at approximately 6 cm in length, weighing only about 5 g [55]. Mortalities during the larval stages have been reported in Australia in *P. chinensis* [56] and India in *P. monodon* [50]. Histopathology lesions in the hepatopancreas include basophilic inclusions within enlarged nuclei of tubule epithelial cells [55]. Like other autonomous parvoviruses, the actively dividing cells (E- and F-cells) at the distal ends of hepatopancreatic tubules show the most HPV inclusions [22,98,114]. The presence of intranuclear inclusion bodies is an important differential diagnosis with other shrimp DNA viruses that produce similar intranuclear inclusions, for example, in mixed infections with DHPV-1 and WSSV (see Figure 2 in Lee et al. [14]). According to Lee et al. [14], in mixed infections with DHPV-1 and WSSV, the presence of basophilic inclusion bodies indicates an advanced stage of WSSV infection, and subsequently, the nuclei disintegrate and their contents fuse with the cytoplasm. In the case of DHPV-1, since the virus does not encode viral DNA polymerase, the inclusion bodies are confined to E-cells, which are known to divide actively [14].

Molecular diagnostic methods (conventional PCR, real-time or quantitative (q)PCR, and in situ hybridization) are instrumental in confirming the etiological role of DPHV-1 in hepatopancreas pathology. PCR methods are useful for screening cultured shrimp for HPV using harmless samples of small appendages or feces and for rapid and easy screening of large numbers of potential hosts and life stages as potential carriers [55]. Both conventional and qPCR primer pairs used for HPV detection are in Table 2. PCR developed by Phromjai et al. [47] was used to confirm HPV propagation in the *Aedes albopictus* cell line C6/36 with the production of cytopathic effects (CPEs) in the form of vacuole formation [97]. Joseph et al. [82] used these primers in nested PCR to screen for HPV in postlarvae of *P. monodon* from hatcheries in India. To overcome the sequence diversity among different strains of DHPV-1 [48,51,104,105,106,107], Srisala et al. [79] developed a universal semi-nested PCR method to detect DHPV-1 in crustaceans by using primer sequences designed from the highly conserved region of the genome. These sequences were also used to make a DIG-labeled probe for in situ hybridization assays to localize DHPV-1 sequences in the histopathology lesions [82]. Cruz-Flores et al. [23] developed cPCR and TaMan qPCR primer pairs and a probe to detect a novel non-pathogenic strain of DPHV-1 in *P. monodon* in Madagascar. They were able to provisionally rule out presence of a non-infectious, endogenous viral element (EVE) [23].

Currently, there is no anti-viral therapy for any viral diseases in shrimp. Therefore, biosecurity and genetically resistant lines remain the cornerstone in managing viral diseases [21]. Gene silencing using the RNAi approach has been reported for both HPV [72] and IHHNV via injection. However, the oral delivery of RNAi molecules remains a challenge, and the utility of RNAi-based therapy has yet to be materialized in shrimp aquaculture [22].

## 4. Genus Penstylhamaparvovirus

The genus *Penstylhamaparvovirus* in the new subfamily *Hamaparvovirinae* with one species, *Decapod penstylhamaparvovirus 1* [1], which is also the type species of its former genus, *Penstyldensovirus*, has four viruses (*Penaeus stylirostris penstyldensovirus* 1 and 2 and *Penaeus monodon penstyldensovirus* 1 and 2). *Penaeus stylirostris penstyldensovirus 1* (PstDV1) is also known as the infectious hypodermal and hematopoietic necrosis virus (IHHNV) [2]. IHHNV was first identified as a pathogen responsible for an economically significant and virulent disease in farmed shrimp (infectious hypodermal and hematopoietic necrosis) in Hawaii, USA, in 1981 [15,16], which led to the collapse of penaeid shrimp farming in the Americas [76]. Phylogenetic analysis revealed the introduction of IHHNV to the American continent in the 1970s in imported *P. monodon* aquaculture stocks from Southeast Asia [115,116]. IHHNV is widely distributed in both wild and farmed shrimps worldwide, including Argentina, Australia, Brazil, the Caribbean, Central America, Ecuador, Indonesia, Israel, Malaysia, New Caledonia, Peru, Philippines, Singapore, Tahiti, Taiwan, Thailand, and the United States of America. The hosts for this virus do not occur in Canada [59].

Based on 24 complete IHHNV genome sequences, the virus has been divided into five genotypes: infectious types I (in Australia), II (in the USA and Southeast Asia), and III (East Asia and Australia), and non-infectious types A (in Madagascar, Australia, Thailand, and India) and B (in Tanzania and Mozambique) [52]. The non-infectious forms of IHHNV are endogenous viral element (EVE) sequences, lacking hairpins [78,117,118], that are inserted into the shrimp host genome following chronic or persistent infection [53,60] and could yield false-positive results for the diagnosis of shrimp infection with IHHNV. These sequences would likely have been eliminated unless they provide beneficial effects, such as EVE-derived immunity (EDI, e.g., mediated by TRIM5a and APOBEC). The IHHNV-EVE has been found to be integrated into chromosome 35 of the *P. monodon* genome, and IHHNV-EVE-related sequences are also present in *P. vannamei* [116,119]. It has been suggested that emerging viruses in shrimp are accommodated through the development of EVE, and after a few years, the infections become subclinical in juveniles [120]. Whether this also occurs in parvovirus infections of vertebrates is not known, but since terrestrial animal parvoviruses might have endogenous parvoviral elements (EPVs) [121], it is tempting to speculate such a mechanism in parvovirus infections in terrestrial animals, particularly humans, where parvovirus infections are mostly subclinical.

WOAH lists IHHNV [61] as an internationally notifiable disease due to its association with significant mortality in Pacific blue leg shrimp (*Penaeus* (*Litopenaeus*) *stylirostris*) and runt deformity syndrome (RDS) in Pacific white shrimp (*Penaeus* (*Litopenaeus*) *vannamei*). The virus is widespread [122] and has been reported in 32 countries in America, Asia, Oceania, and Africa [52,62,122,123,124]. In addition, it has been detected in about 30 species of shellfish, including in wild and farmed penaeid shrimps *P. stylirostris* [115], *P. vannamei* [125,126], *P. monodon* [127,128], hybrid penaeid shrimp (*P. esculentus* hybridized with *P. monodon*) [62], and several others [52]; in non-penaeid shrimp *Macrobrachium rosenbergii* [46,129]; in crayfish *Procambarus clarkii* [130,131] and *Cherax quadricarinatus* [132]; in crabs *Hemigrapsus penicillatus*, *Neohelice granulate*, *Callinectes arcuatus*, and *Sesarma reticulatum* [123,133,134,135]; and in bivalve shellfish [136].

A previously identified shrimp parvovirus, spawner-isolated mortality virus (SMV), and its corollary lymphoidal parvovirus [137] were abolished, and the science transferred to IHHNV and *Carnobacterium divergens* after it was shown that the SMV sequence (GenBank Accession # AF499102.1) was that of *C. divergens* (Accession # CP016843.1) [138]. Thus, the host range of IHHNV now includes redclaw crayfish *Cherax quadricarinatus* in northern Australia [139]. According to Owens 2023 [138], this publication [139] is now discarded except for the description of stress-related deaths, and the limited data are transferred to IHHNV.

**Figure 7:**
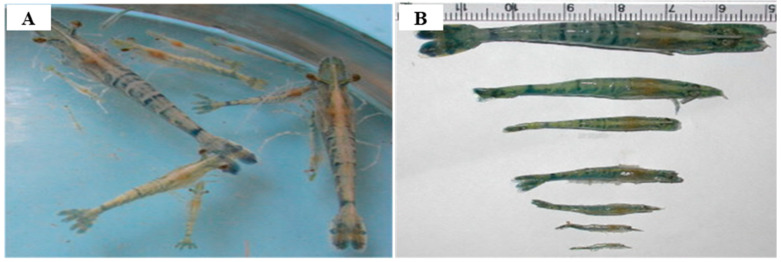
Size variations observed in 50-day-old *Penaeus monodon* with infectious hypodermal and hematopoietic necrosis virus (IHHNV) (**A**,**B**). (Reprinted from *Aquaculture*, Vol. 289 (3–4), Rai, P., Size variations observed in 50-day-old *Penaeus monodon* with infectious hypodermal and hematopoietic necrosis virus (IHHNV) (**A**,**B**). Reproduced from [146], Figure 10, under Creative Commons Attribution License (https://creativecommons.org/licenses/by/4.0/ (accessed 21 December 2023))).

IHHNV is still an important virus threatening shrimp aquaculture [52,124] even though genetically resistant shrimp populations have been developed [17,61]. IHHNV infection causes an acute disease with 80–100% mortality in postlarvae and juveniles of *P. stylirostris* [60] and postlarvae of *M. rosenbergii* [46]. The affected animals stop swimming, tumble, and then slowly sink to the bottom of the pond, and usually, they are ingested by healthy shrimps [140]. Very high mortality was reported in experimental IHHNV infection of crayfish *P. clarkii* (19/20 animals died) [130]. IHHNV infection causes chronic disease, runt deformity syndrome (RDS), characterized by slow growth and deformities in the exoskeleton such as a bent (45° to 90° bend to left or right) or otherwise deformed rostrum, a deformed sixth abdominal segment, wrinkled antennal flagella, cuticular roughness, “bubble-heads,” and other cuticular deformities, without mortality, in juveniles of *P. vannamei* and *P. monodon* (Figure 7) [13,24,61,78,126,141], leading to 50% of the economic loss of the shrimp industry [142]. IHHNV infection can cause 30–90% growth retardation in juvenile *P. vannamei* [13,117]. Microscopic lesions include prominent intranuclear, Cowdry type A inclusion bodies characteristic of parvoviruses. The inclusion bodies observed in IHHNV occur in tissues of the ectodermal epithelium of the fore- and hindgut, and they have mesodermal origins like the hematopoietic organs, antennal gland, and lymphoid organ [143]. Like other parvoviruses, IHHNV targets rapidly multiplying host cells, hence the severe infection of younger shrimp [117]. Adults of *P. vannamei* [144] and non-penaeid shrimp such as *M. rosenbergii* [52,129] are carriers of IHHNV without apparent clinical disease. Crayfish *P. clarkii* is also asymptomatic when naturally infected by IHHNV [131,145]. Red claw crayfish *C. quadricarinatus* could be a potential carrier of the virus [132], and crabs and bivalve shellfish can be asymptomatic carriers of IHHNV [52].

White spot syndrome virus (WSSV) is a differential diagnosis for IHHNV [141]. IHHNV has been reported to interfere with WSSV during mixed infection in penaeid shrimp, leading to higher survival rates compared to infection with WSSV alone [52,116,147,148]. Analysis of genetic diversity among IHHNV isolates in the Gulf of California found an unexpectedly high mutation rate that was comparable to that reported for RNA viruses, suggesting the potential for a new virulent strain to arise that might lead to epizootics similar to those observed in the early 1990s [149].

The preferred molecular method for diagnosing shrimp infection with IHHNV is conventional and or quantitative (q)PCR for which several PCR primer pairs (see Table 2) have been published, including those developed by Nunan et al. [88] that amplify 40% of the viral genome; by Tang et al. [94] and Rai et al. [95] that are recommended WOAH [61] to specifically detect infectious IHHNV forms and exclude the noninfectious related sequences; and by Dhar et al. [93] and Cowley et al. [96] that are used for molecular detection of IHHNV by qPCR with SYBR Green detection and TaqMan RT-qPCR, respectively. The SYBR Green qPCR was 2000-fold more sensitive than the conventional PCR [93], but this test design did not consider IHHNV non-replicating endogenous viral element (EVE) sequences. A second TaqMan RT-qPCR developed by Cowley et al. [96] specifically detected IHHNV-EVE sequences if also present in the sample. More recently, Arbon et al. [150] reported screening for six viral pathogens, including IHHNV, in Australian wild-sourced giant black tiger shrimp (*Penaeus monodon*) broodstock using the IHHNV TaqMan qPCR assay of Cowley et al. [96]. Arbon et al. [150] used TaqMan qPCR analysis to determine tissue-specific loading of IHHNV to support the selection of appropriate diagnostic samples. The highest viral load was in gill tissue, followed sequentially by the hindgut, pleopod, hepatopancreas, lymphoid organ, ventral nerve cord, and abdominal muscle [150].

Sequences of the whole IHHNV genome can be obtained using conventional PCR and the eight specific primers designed by Silva et al. [151]. In situ hybridization using IHHNV-specific DNA probes such as BA402 (available in kit form from DiagXotics Inc., 27 Cannon Rd., Wilton, CT 06897, USA) can be used on tissue sections preserved in Davidson’s, AFA, or formalin [59]. A recombinase polymerase amplification (RPA) combined with a lateral flow dipstick (LFD) was developed for detecting PstDV (IHHNV) [152]. This protocol had reduced false positives due to IHHNV-EVE than the conventional PCR protocol of Tang et al. [94] (5% compared to 76-78%) [152]. Recently, a probe-based real-time enzymatic recombinase amplification (ERA) system was reported to rapidly detect IHHNV in *P. vannamei* [150]. The IHHNV-ERA could amplify infectious IHHNV but not common EVE sequences [153].

IHHNV infection control and prevention are achieved through stocking high-quality disease-free seeds, optimum rearing conditions, and good farm management practices [142].

## 5. Genus Ichthamaparvovirus

The genus *Ichthamaparvovirus* in the new subfamily *Hamaparvovirinae* (Figure 2 and Figure 4) has at least two species, *Syngnathid ichthamaparvovirus 1* (*Syngnathus scovelli* chapparvovirus) (the type species) and tilapia parvovirus (TiPV). *Syngnathus scovelli* chapparvovirus has partially sequenced hairpins, suggesting that the genus is heterotelomeric [1].

### 5.1. Syngnathid Ichthamaparvovirus 1

The near-complete genome sequence of *Syngnathid ichthamaparvovirus* 1 was identified in previously unreported DNA sequences by screening published whole genome sequencing (WGS) data of a gulf pipefish’s homogenized gill, muscle, and male brood pouch tissue (*Syngnathus scovelli*) [1,58]. Therefore, it is unknown if this virus is associated with clinical disease. In a different syngnathid fish (family *Syngnathidae*), the tiger tail seahorse (*Hippocampus comes*), an endogenous viral element (EVE) with 70% NS1 amino acid sequence identity to *Syngnathid ichthamaparvovirus 1* was found [58].

### 5.2. Tilapia Parvovirus (TiPV)

**Figure 8:**
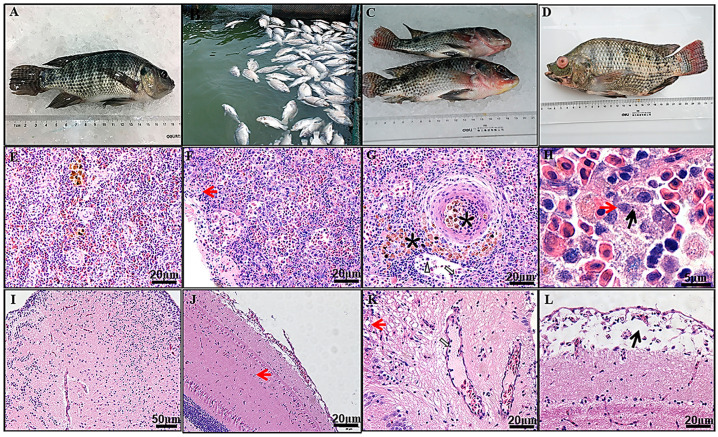
Outbreaks, clinical symptoms, and pathological analysis. (**A**) Healthy tilapia; (**B**) tilapia disease outbreak in cage-cultured results in massive mortality (August 2015; Jinmen, Hubei province, China); (**C**,**D**) gross pathological signs of infected tilapia, including hemorrhages on the lower jaw, anterior abdominal, and the fin bases, accompanied by exophthalmos eyes and pronounced ocular lesions. (**E**) Healthy spleen; (**F**) moderated diseased spleen infected on the 3rd day post TiPV infection; (**G**,**H**) severe diseased spleen infected on the 5th day post TiPV infection. Lymphocytes (white arrow) and macrophages (white triangular arrowheads) in diseased spleen sinusoids, necrotic splenocytes (red arrow), virus inclusion body (black arrow), and melanomacrophage centers (asterisk) in affected spleen; (**I**) healthy brain; (**J**) moderate diseased brain infected on the 3rd day post TiPV infection, vacuolated neurons with marginated nucleus (red arrow); (**K**,**L**) severe diseased brain infected on the 5th day post TiPV infection, vacuolated neurons with marginated nucleus (red arrow), lymphocytes (white arrow) in the blood vessel, edema of cerebral cortex (black arrow). HE staining. Bar = 20 um (**A**,**B**,**E**,**F**), 50 um (**D**), 5 um (**C**). (Reproduced under Creative Commons Attribution License (https://creativecommons.org/licenses/by/4.0/ (accessed 21 December 2023)) from [25], Figure 1).

**Figure 9:**
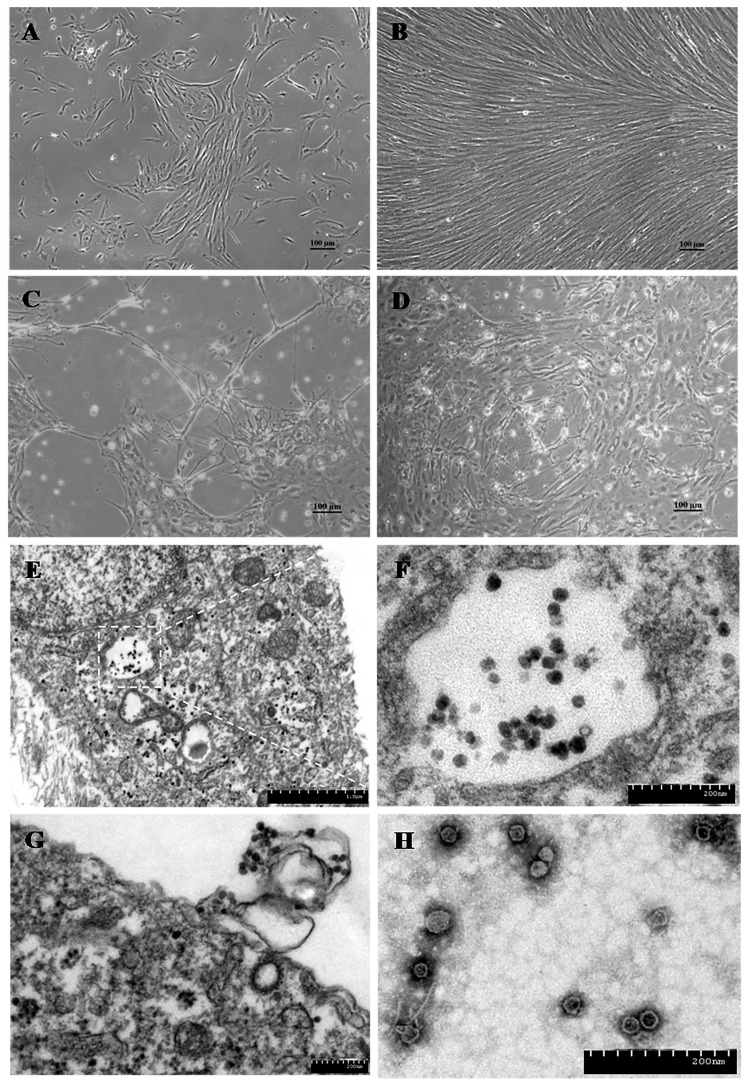
Morphology of the tilapia brain cells (TiBs) and cytopathic effect (CPE) induction on TiBs induced by TiPV and transmission electron micrographs of the TiPV-infected TiB cells. (**A**) The TiB cells at passage 1, 10 days; (**B**) the TiB cells at passage 2, 3 days; (**C**) TiB cells infected with TiPV at passage 3, 5 days post infection; (**D**) TiB cells infected with TiPV at passage 6, 3 days post infection (bar = 100 μm). (**E**) Virus particles existed in the cytoplasm and nuclei (white arrow), Nu: nucleus. (bar, 1 μm); (**F**) high magnification of the region in the white rectangular box indicated in Panel A, virus particles aggregated in the cytoplasm (bar, 200 nm); (**G**) the virus releasing at the plasma membrane of the TiB cell (bar, 200 nm); (**H**) purified TiPV particles negatively stained with 2% phosphotungstic acid (bar, 200 nm). (Reproduced under Creative Commons Attribution License (https://creativecommons.org/licenses/by/4.0/ (accessed 21 December 2023)) from [25], Figure 2).

Tilapia parvovirus (TiPV) is an emerging pathogen in tilapia aquaculture, detected in China, Thailand, and India. Clyde et al. [154] have reviewed all the current viral infections reported in tilapia, including TiPV, TiLV, tilapia larvae encephalitis virus (TLEV), infectious spleen and kidney necrosis virus (ISKNV), Bohle iridovirus (BIV), lymphocystis disease virus (LCDV), infectious pancreatic necrosis virus (IPNV), nervous necrosis virus (NNV), and iridovirus-like agent. TiPV was first identified using next-generation sequencing (NGS) on fecal samples from crocodiles in China fed with tilapia [29]. Further investigation using a novel semi-nested PCR assay with primers targeting the viral NS1 gene detected the virus in tilapia intestine samples [29]. TiPV was subsequently associated with mass morbidity and 60–70% mortality in China’s farmed adult Nile tilapia (500–600 g) [25]. Clinical signs in affected fish included lethargy, anorexia, change in swim behavior, multifocal hemorrhage, and ocular lesions [25]. Microscopic lesions included splenic necrosis, encephalitis, nephritis, hepatitis, and gill branchitis [25] (Figure 8). Virions consistent with the parvovirus morphology were seen by electron microscopy in the cytoplasm and nucleus of cells of the heart, spleen, kidneys, brain, gills, and intestine [25]. The kidney and spleen were positive for ISH, PCR, and IFA [25]. The TiPV was isolated and propagated in tilapia brain cells (TiBs) and induced a typical cytopathic effect (CPE) after three days post-infection (dpi) (Figure 9). This virus was used to infect adult tilapia experimentally, and clinical disease symptoms similar to those observed naturally were replicated [25]. In a more recent case of TiPV infection in adult Nile tilapia in Thailand, Dong et al. [155] reported Cowdry type A inclusion bodies in acinar cells of the pancreas as a diagnostic histopathological feature (i.e., pathognomonic of TiPV infection), which is also diagnostic of other parvoviral infections in shrimp and terrestrial species [155].

Recently, TiPV has been reported in mixed infections with tilapia lake virus (TiLV), family *Amnoonviridae*, and polymicrobial infections in farmed juvenile red hybrid tilapia in Thailand [26,27], and in farmed Nile tilapia (*Oreochromis niloticus*) in India [156]. Dong et al. [155] reported TiPV in mixed infection with *Streptococcus agalactiae* in adult Nile tilapia in Thailand. In the disease outbreak investigated by Piewbang et al. [27], the mortality rate among farms reached 50–75%, with the most affected fish weighing 10–30 g, and the lesser affected fish weighing 300–800 g. In the disease investigation reported by Yamkasem et al. [26], it was noted that differential diagnosis of TiPV and TiLV was difficult because there were no pathognomonic clinical signs, and no fish was found to be infected with TiPV alone. Moreover, the Thailand TiPV isolate (strain KU01-TH/2020’ (MW685502)) had a sequence identity of 98.74% to the virus first isolated in China (MT393593) [26]. In the report by Rajendran et al. [156], in one geographical region in India, TiPV was detected along with TiLV and/or *Aeromonas* spp., whereas, in another region, fish were apparently healthy, and only TiPV could be detected in the fish samples.

Molecular techniques, particularly conventional PCR and qPCR are now commonly used to detect viral pathogens in tilapia aquaculture [155]. Table 2 lists the primer and probe sequences and the size of the PCR products used in the following protocols for the detection of TiPV in tilapia tissue samples. Liu et al. [25] reported primers for the detection of TiPV by conventional PCR and qPCR with SYBR Green detection targeting across the NS1, NP, and NS1 gene regions, respectively (Table 2). Analysis of TiPV distribution and viral loading in nine different tissues of naturally infected tilapia using qPCR showed the highest genome copies in the kidney (3.5 × 10^7.32±0.21^/mg) and spleen (4.2 × 10^7.12±0.35^/mg) followed by the intestine (4.3 × 10^6.37±0.36^/mg), heart (5.1 × 10^6.25±0.27^/mg), and brain (1.8 × 10^6.09±0.24^/mg), with the lowest genome copies in the gill (1.8 × 10^4.32±0.17^/mg), liver (3.5 × 10^4.15±0.26^/mg), and eye (2.8 × 10^3.78±0.32^/mg) [25]. In addition, PCR revealed the prevalence of TiPV infection in six cities across three provinces in China, ranging from 22.6% to 64.6% [25]. Yamkasem et al. [26] reported a PCR protocol for TiPV in farmed red hybrid tilapia naturally coinfected with TiLV in Thailand, and Prasart and Surachetpong [157] used it to detect TiPV with a multiplex PCR protocol for the simultaneous detection of three viral pathogens including TiPV (in fish tissue samples collected from moribund and healthy fish from tilapia farms in central and western Thailand and Ghana). More recently, Yamkasem et al. [100] reported TaqMan RT-qPCR assays targeting the NS1 and VP1 genes of TiPV. When used on farmed red hybrid tilapia in Thailand, these TaqMan qPCR assays had comparable sensitivity to the qPCR with SYBR Green detection [25] but were more sensitive (100–1000-fold more sensitive) than the conventional PCR assays [25,26,100] although they were not tested on TiPV from other geographical locations [100]. Zhao et al. [101] developed a sensitive droplet digital PCR (ddPCR)-based assay to rapidly detect and quantify TiPV for the surveillance of sources and transmission routes of TiPV. The ddPCR used the same primers and probe as qPCR, and ddPCR was 60 times more sensitive than qPCR; the detection limit of ddPCR and qPCR were 0.07 and 4.63 copies/μL, respectively [101]. The spleen is the best tissue for detecting TiPV by PCR in apparently healthy tilapia [156]. Phusantisampan et al. [158] developed a loop-mediated isothermal amplification (LAMP) method for rapid and simple screening and detection of TiPV in tissue samples from infected tilapia. The TiPV LAMP method used six primer sets targeting the conserved sequences of the NS gene, which is conserved among *Parvoviridae* and thus targeted for most molecular detection assays [5,25]. When applied to clinical samples, the TiPV LAMP assay showed no false-positive results, and the false-negative interpretations were low, demonstrating that it could be applied to screen for TiPV infection on tilapia farms [158].

The high pathogenicity of TiPV (highly fatal to adult tilapia [25,28]) dictates that its host range must be determined and adequate control measures developed against TiPV disease in tilapia aquaculture.

## 6. Unassigned

### 6.1. Metalloincertoparvovirus

*Metalloincertoparvovirus* is an undefined genus (Figure 2) that contains one species, *Metalloincertoparvovirus decapod 1*, with the virus *Penaeus monodon* metallodensovirus (PmMDV). PmMDV was derived from a mass mortality event affecting *P. monodon* in Vietnam [30]. The genome of PmMDV is 4374 nt in length (GenBank accession # MK028683), flanked by ITRs of 416 nt, of which 161 nt fold into a regular, T-shaped hairpin, and it has a unique genome organization and transcription strategy encoding up to eight gene products [30]. All other parvovirus genera have 3-7 gene products [29]. Although the PmMDV NS and VP genes lack significant similarity to those of any members of the *Parvoviridae*, the PmMDV NS1 protein still harbors the SF3 helicase domain. Furthermore, its capsid structure displays T = 1 icosahedral symmetry, consistent with all members of the *Parvoviridae* thus far. Therefore, PmDV fulfills all criteria to be classified as a parvovirus, although it does not cluster within any of the currently established subfamilies. The ICTV decided not to classify it under any existing subfamilies (Figure 3) [31].

### 6.2. Crangon Crangon Parvo-Like Virus (CcPaLV)

*Crangon crangon* parvo-like virus (CcPaLV) was discovered in a virome study of the European brown shrimp (*Crangon crangon*) using NGS [7]. That study described near-complete genomes of 16 novel viruses, most of which were distantly related to unclassified viruses or viruses belonging to the *Picornavirales*, *Bunyavirales*, *Nudiviridae*, *Parvoviridae*, *Flaviviridae*, *Hepeviridae*, *Tombusviridae*, *Narnaviridae*, *Nodaviridae*, and Sobemovirus. In addition, the study observed a difference in virome composition between muscle and hepatopancreatic tissue, suggesting a distinct tissue tropism of several of these viruses [7].

CcPaLV is highly divergent from known members of the subfamily *Parvovirinae*; it clusters between the subfamilies *Parvovirinae* and *Densovirinae*, although it is slightly more related to the subfamily *Parvovirinae* [7]. Whether this novel virus represents a novel subfamily in the family *Parvoviridae* or a very distinct genus in the subfamily *Parvovirinae* [7] remains to be seen.

### 6.3. Novel Salmon Parvovirus from Sockeye Salmon

A full genome sequence of a novel salmon parvovirus—the first parvovirus to be identified in a fish species—was obtained from sockeye salmon smolts from British Columbia, Canada. However, it is unknown whether it is associated with disease [6]. The novel salmon parvovirus was detected using NGS for virus discovery. It was found in a high load in sockeye salmon smolts migrating to the ocean, with load and prevalence generally declining from summer to fall. Prevalence varied greatly among stocks and years [6]. The full genome sequence of this novel salmon parvovirus is not publicly available, and its taxonomic location in the *Parvoviridae* family remains unassigned.

### 6.4. Novel Zander Parvovirus

**Figure 10:**
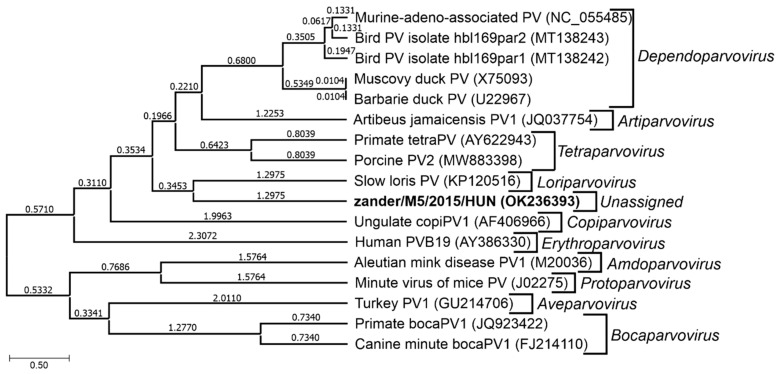
Phylogenetic analysis of zander/M5/2015/HUN (OK236393, bold letters) and representatives of 10 genera in the subfamily *Parvovirinae* based on the ~460 aa long tripartite helicase domain of NS1. The dendrogram was constructed based on an amino acid sequence alignment of tripartite helicase domains by Bayesian Evolutionary Analysis Utility version v1.10.4 (BEAST) [160] using the LG + I + G + F substitution model, a lognormal relaxed clock, and Yule process, throughout 10,000,000 generations. The tree is drawn to scale with branch lengths measured in the average number of substitutions per time unit. PV = parvovirus. (Reproduced under Creative Commons Attribution License (https://creativecommons.org/licenses/by/4.0/ (accessed 21 December 2023)) from [5], Figure 2).

Novel zander parvovirus (zander/M5/2015/HUN, OK236393) from zander or pikeperch (*Sander lucioperca*), a freshwater fish in Hungary, potentially represents a new genus and a new species in the subfamily *Parvovirinae* [5] (Figure 10). The novel zander parvovirus was detected by viral metagenomics and PCR methods in three out of seven (42.8%) fecal samples collected from fish showing no clinical signs [5]. While the novel zander parvovirus may be the third member in the subfamily *Parvovirinae* to infect an aquatic animal host, its origin, whether from fish tissues or the fish diet, was not established [5]. Furthermore, one of the positive zander fecal samples also contained a potentially novel fish-origin picornavirus, the family *Picornaviridae* [159], as a coinfection [5].

A sequence-specific screening PCR primer pair (Table 2) targeting the NS1 region of the viral genome has been reported [5].

### 6.5. Spawner-Isolated Mortality Virus (SMV)

Spawner-isolated mortality virus (SMV) was initially reported to belong to the family *Parvoviridae* and to cause mortalities in broodstock of *Penaeus monodon* with mid-crop mortality syndrome on grow-out farms in northern Australia [161,162,163] and redclaw crayfish *Cherax quadricarinatus* in northern Australia [139]. However, recent sequence analysis of the SMV entity has shown it to be *Carnobacterium divergens* [138]. Therefore, the publications about SMV have been assessed and it was recommended to abolish its name with the still valid science transferred to IHHNV and *C. divergens* [138].

## 7. Concluding Remarks

This review shows that the pathogenesis of aquatic animal (finfish, crustaceans, and mollusks) parvovirus diseases is comparable to that of terrestrial animal parvovirus diseases. These viruses cause diseases that range from subclinical to lethal, such as mass morbidity and mortality in naïve animal populations, particularly in young (juvenile) animals. Virus replication takes place in the nucleus, hence the characteristic intranuclear inclusion bodies, and requires host cell functions of the late S phase or early G2 phase of the cell division cycle, a requirement for cycling cells that is the basis for many aspects of the pathogenesis of parvovirus infections. Because of their small genome, parvoviruses target tissues with a high cell turnover (i.e., autonomous parvoviruses) or require coinfection with helper viruses (i.e., defective parvoviruses). Moreover, the differences in the immune system of aquatic animals compared to terrestrial animals dictate that vaccination is not as effective in controlling aquatic animal parvoviruses as it is for terrestrial animal parvoviruses; in fact, it is not an option in crustaceans and mollusks. Fish vaccine development and availability is one of the major issues in aquaculture, in addition to biosecurity protocols, genomic surveillance, and advances in diagnostics, whereas implementing disease-free stocks is a big concern in shrimp aquaculture. The shrimp parvoviruses PstDV1/IHHNV, DPHV-1/HPV, and PmMDV are responsible for major economic losses in shrimp aquaculture. Still, while IHHNV is on the WOAH list of reportable diseases, HPV was de-listed in 2012, and there is a lack of information about PmMDV. Interestingly, shrimp accommodate emerging viruses such as IHHNV through the development of EVE, and after a few years, the infections become subclinical in juveniles. Whether this also occurs in parvovirus infections of vertebrates is not known, but it is tempting to speculate such a mechanism in parvovirus infections in terrestrial animals, particularly humans, where parvovirus infections are mostly subclinical. The fish parvovirus TiPV is an emerging pathogen associated with massive morbidity and mortality in tilapia, leading to extensive losses in the burgeoning aquaculture industry. TiPV, which was first reported in 2019, has not yet been listed by the WOAH. How many viral species in the *Parvoviridae* family will affect aquaculture economically is still unknown. Additionally, mass mortalities in sunflower sea stars and freshwater mussels underscore the importance of densoviruses in wild aquatic organisms.

## Figures and Tables

**Table 2:** Virus isolation and propagation and molecular detection by PCR/RT-PCR of aquatic animal parvoviruses.

Virus Subfamily	Virus Genus	Virus Species or Isolate ^1^ in Host		Virus Isolation and Propagation		Conventional PCR/RT-PCR or Quantitative (q)PCR/RT-PCR	
		Fish	Crustaceans	Cell Lines ^2^	Incubation Temp. ^3^	Virus-Specific Primers/Probes	Amplicon Size, PCR Chemistry ^4^, and Reference
*Densovirinae*	*Aquambidensovirus*		CqDV SSaDV	- -	- -	CqDV 5 Fq 5′-CGCTGTGGAGAGTGCACTAGAGGC-3′ 2A Rq 5′-TCTGAATCAATCTCCTCACGATCGC-3′ VP1 forward primer 5′-TGGCCACTCATCATGTCTCT-3′ VP1 reverse primer 5′-CTTGGGGTCCTTCATGAGC-3′	283 bp, qPCR-SYBR Green [89] 534 bp, cPCR [91]
*Hamaparvovirinae* *Parvovirinae*	*Penstylhamaparvovirus* *Hepanhamaparvovirus* *Ichthamaparvovirus* Unassigned	TiPV Novel zander parvovirus	IHHNV HPV/DHPV-1	- C6/36 ^6^ E-11; TiB	- 28 °C 25 °C 28 °C	77012F 5′-ATCGGTGCACTACTCGGA-3′ 77353R 5′-TCGTACTGGCTGTTCATC-3′ 313F Forward 5′-AGGAGACAACCGACGACATCA-3′ 363R Reverse 5′-CGATTTCCATTGCTTCCATGA-3′ IHHNV309F 5′-TCCAACACTTAGTCAAAACCAA-3′ IHHNV309R 5′-TGTCTGCTACGATGATTATCCA-3′ IHHNV648F 5′-GAACGGCTTTCGTATTTTGG-3′ IHHNV648R 5′-AGCGTAGGACTTGCCGATTA-3′ 77112F 5′-ATCGGTGCACTACTCGGA-3′ 77012R 5′-′TCGTACTGGCTGTTCATC-3′ 389F 5′-CGGAACACAACCCGACTTTA-3′ 389R 5′-CGCCAAGACCAAAATACGAA-3′ IHHNV-q309F1 5′-CCTAAAGAAAACAGTGCAGAATAT-3′ IHHNV-q309R1 5′-TCATCGTCAAGTTTATTGACAAGTTC-3′ IHHNV-q309Pr1 5′-6FAM-CTCCAACACTTAGTCAAA-TAMRA-3′ HPVF 5′-GCATTACAAGAGCCAAGCAG-3′ HPVR 5′- ACACTCAGCCTCTACCTTGT-3′ qHPVF 5′- CGCGGATCCAGGTAGAGGCTGAGTGTAA-3′ qHPVR 5′-CGCGAATTCCAGGTAGTGACGCCGAAA-3′ 1120F 5′-GGTGATGTGGAGGAGAGA-3′ 1120R 5′-GTAACTATCGCCGCCAAC-3′ HPVnF 5′-ATAGAACGCATAGAAAACGCT-3′ HPVnR 5′-CAGCGATTCATTCCAGCGCCACC-3′ DHPV-U 1538 F 5′-CCTCTTGTTACATTTTACTC-3′ DHPV-U 1887 R 5′-GATGTCTTCTGTAGTCC-3′ DHPV-U 1622 F 5′-AAGTTTGCACAGTGGTTGT-3′ HPV F 5′- CCACAACATAAGTGCTGCAGT-3′ HPV R 5′- TAGCCGCGGAATAAAACCCT-3′ TaqMan probe for HPV Madagascar 5′- 6FAM -TGAATGTTGTAAAGACTCAGCCA- TAMRA-3 HPV F 5′-AAGCCTGTGTTTCTGACTAACCA-3′ HPV R 5′- TGAGTTTACCGCCTCACTTCC-3′ TiPV-Fq 5′-GAGATGGTGTGAAAATGAACGGG-3′ TiPV-Rq 5′-CTATCTCCTCGTTGCTCGGTGTATC-3′ TiPV-F 5′-GAGATGGTGTGAAAATGAACGGG-3′ TiPV-R 5′-CTATCTCCTCGTTGCTCGGTGTATC-3′ VP734F 5′-TGGCTTTATGGACTTTGCTGAT-3′ VP734R 5′-CATCCCTCCTGCTCTTGGTT-3′ TiPV-NS124F 5′-AAAGTACCTAAGGCGGAGCG-3′ TiPV-NS124R 5′- TTTCAATCACCTTCCCGCCA-3′ TiPV-NS124Probe 5′-6FAM-CCGAAGAGCATAGTGG-BHQ1-3′ TiPV-VP205F 5′-CCAGATTGAAAGGGGCACGA-3′ TiPV-VP205R 5′-TTGGTGTTGGTGGTACGCAT-3′ TiPV-VP205Probe 5′-6FAM-CCAGTCCCGACCTACTCAAA-BHQ1-3′ TiPV-NS965F 5′-TGGCTACCGAGAAGGGGTTA-3′ TiPV-NS965R 5′-GCTCTTCCCGCTTGAGTCTT-3′ TiPV Forward 5′-GTATTAGTGGCGTCATTGCAGAG-3′ TiPV Reverse 5′-GGCAGGTTCCCCACTTCAC-3′ TiPV Probe 5′-6FAM-CCCTTCTCGGTAGCCAC-MGB-3′ ZanderParvoscreen-F 5′-GGCTAATCATCAAACAGGAAAGA-3′ ZanderParvo-screen-R 5′-AGCTCC CACCACTTAATATCTT-3′	1681 bp, cPCR [92] 50 bp, qPCR-SYBR Green [93] 309 bp, cPCR [94] 648 bp, cPCR-1st step ^5^ [95] 356 bp, cPCR [82] 389 bp, cPCR [82] 98 bp, qPCR -TaqMan [96] 441 bp, cPCR [47,97] 147 bp, qPCR-SYBR Green [97] 592 bp, cPCR [98] 265 bp, cPCR-2nd step ^7^ [99] 350 bp and 266 bp, cPCR-semi nested [51] 165 bp, cPCR and TaqMan [23] 120 bp, cPCR [23] 134 bp, qPCR-SYBR Green [25] 534 bp, cPCR [25] 734 bp, cPCR [26] 124 bp, qPCR-TaqMan [100] 205 bp, qPCR-TaqMan [100] 965 bp, cPCR [100] ddPCR [101] 492 bp, cPCR [5].

^1^ Virus abbreviations: CqDV = *Cherax quadricarinatus* densovirus; SSaDV = sea star-associated densovirus; IHHNV = infectious hypodermal and hematopoietic necrosis virus; HPV = hepatopancreatic parvovirus/DHPV-1 = *Decapod hepanhamaparvovirus 1*; TiPV = tilapia parvovirus. ^2^ Cell line abbreviations: E-11 = cloned from SSN-1 cell line; TiB = tilapia brain; C6/36 = *Aedes albopictus* cell line. ^3^ Fish viruses are typically isolated on cultured fish cell lines at 15–25 °C. ^4^ PCR chemistry: conventional PCR = cPCR; real-time or quantitative (q)PCR = qPCR, either SYBR Green or TaqMan. ^5^ These are nested PCR primers; the 2nd step primers are IHHNV309F/ IHHNV309R of Tang et al. [94]. ^6^ HPV/DHPV-1 was propagated in the *Aedes albopictus* cell line C6/36 with the production of cytopathic effects (CPEs) in the form of vacuole formation [97]. ^7^ These are nested PCR primers; the 1st step primers are HPVF/HPVR of Phromjai et al. [47].

## Data Availability

The original contributions presented in the study are included in the article, further inquiries can be directed to the corresponding author.
